# Detecting *TP53* mutations in paired liquid and tissue biopsies from patients with high‐grade serous ovarian carcinoma

**DOI:** 10.1002/ijc.70277

**Published:** 2025-12-09

**Authors:** Amanda Olsson Widjaja, Peter Micallef, Maria Lycke, Tobias Österlund, Manuel Luna Santamaría, Julia Hedlund Lindberg, Therese Carlsson, Ulf Gyllensten, Anders Ståhlberg, Benjamin Ulfenborg, Anna Linder, Karin Sundfeldt

**Affiliations:** ^1^ Sahlgrenska Center for Cancer Research, Department of Obstetrics and Gynecology, Institute of Clinical Sciences, Sahlgrenska Academy University of Gothenburg Gothenburg Sweden; ^2^ Sahlgrenska Center for Cancer Research, Department of Laboratory Medicine, Institute of Biomedicine, Sahlgrenska Academy University of Gothenburg Gothenburg Sweden; ^3^ Wallenberg Centre for Molecular and Translational Medicine University of Gothenburg Gothenburg Sweden; ^4^ Region Västra Götaland, Department of Clinical Genetics and Genomics Sahlgrenska University Hospital Gothenburg Sweden; ^5^ Department of Immunology, Genetics, and Pathology, Biomedical Center, SciLifeLab Uppsala Uppsala University Uppsala Sweden; ^6^ Sahlgrenska Center for Cancer Research, Department of Medical Chemistry and Cell Biology, Institute of Biomedicine, Sahlgrenska Academy University of Gothenburg Gothenburg Sweden; ^7^ Systems Biology Research Center, Department of Biology and Bioinformatics, School of Bioscience University of Skövde Skövde Sweden; ^8^ Region Västra Götaland Sahlgrenska University Hospital Gothenburg Sweden

**Keywords:** ctDNA, liquid biopsy, next generation sequencing, ovarian cancer biomarkers, UMI

## Abstract

High‐grade serous ovarian carcinoma (HGSC) is the most lethal form of ovarian carcinoma, often diagnosed at advanced stages due to non‐specific symptoms and the lack of reliable screening methods. This proof‐of‐concept study aimed to develop a robust *TP53* mutation panel for detecting HGSC through targeted DNA sequencing in both liquid and solid biopsies. We constructed a custom *TP53* gene panel and utilized a PCR‐based unique molecular identifier approach for next‐generation sequencing to analyze 94 samples from 11 patients diagnosed with HGSC, including primary tumor, plasma, ascites, ovarian cyst fluid, vaginal, endocervical and endometrial samples. Detected *TP53* mutations were analyzed, categorized, and their frequencies calculated. Pathogenic *TP53* mutations were identified in all patients, with 91% of the patients exhibiting one unique paired mutation across three or more sample types. The panel demonstrated high sensitivity and technical reproducibility, successfully detecting *TP53* mutations across all sample types, with as little as 2.6 ng of DNA. *TP53* mutations were consistently found in ascites, ovarian cyst fluid, and plasma samples, confirming the presence of pathogenic mutations in each sample type across all patients. This study underscores the potential of liquid biopsies in clinical molecular diagnostics. The *TP53* mutation panel presented in this proof‐of‐concept study offers a promising approach for differential diagnostics and detection of HGSC, informative data prior to extended investigation and first‐line treatment guidance.

## What's New?

High‐grade serous ovarian carcinoma is often diagnosed at advanced stages due to non‐specific symptoms and the lack of reliable screening methods. This proof‐of‐concept study introduces a novel *TP53* mutation panel using unique molecular identifier‐based next‐generation sequencing for sensitive detection of high‐grade serous ovarian carcinoma in liquid and solid biopsies. With minimal DNA input (as little as 2.6 ng), the same pathogenic mutation was identified in multiple sample types from each patient. The findings highlight the potential of non‐invasive liquid biopsies for high‐grade serous ovarian carcinoma detection, offering a promising tool in clinical molecular diagnostics and treatment decision‐making.

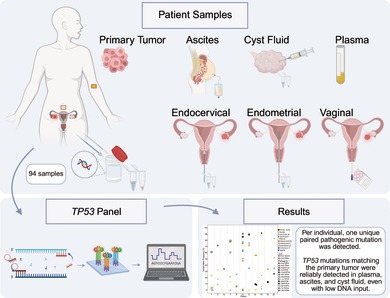

**Abbreviations**
BMI: Body mass index
CA‐125: Serum cancer antigen‐125
cfDNA: Cell‐free DNA
COSMIC: Catalogue of somatic mutations in cancer
CT: Computer tomography
ctDNA: Circulating tumor‐DNA
FFPE: Formalin‐fixed and paraffin‐embedded
FIGO: International Federation of Gynecology and Obstetrics
HGSC: High‐grade serous ovarian carcinoma
LB: Liquid biopsy
NGS: Next‐generation sequencing
OC: Ovarian carcinoma
S: Solid cell pellet
SiMSen‐seq: Simple, multiplexed, PCR‐based barcoding of DNA for sensitive mutation detection using sequencing
STIC: Serous tubal intraepithelial carcinoma
*TP53*
: tumor protein p53
UMI: Unique molecular identifier
VAF: Variant allele frequency
VUS: Variant of uncertain significance

## 1 INTRODUCTION

High‐grade serous ovarian carcinoma (HGSC) accounts for almost 70% of all ovarian carcinoma (OC)‐associated deaths, primarily due to absent or non‐specific symptoms.^1^ The need for diagnostic methods capable of detecting OC is unmet, particularly for this aggressive histotype. The most frequent genetic aberrations in HGSC are mutations in the Tumor protein p53 (*TP53*) gene, detected in more than 90% of cases.^2^, ^3^

Our understanding of OC pathogenesis has advanced with the finding that HGSC mainly originates from a precursor lesion in the fallopian tube fimbriae, known as Serous Tubal Intraepithelial Carcinoma (STIC).^4^ Women diagnosed with concurrent HGSC in the ovary and STIC lesions have been found to possess identical *TP53* mutations,^5^ suggesting that this shared genetic aberration can be used for detection of HGSC already at a premalignant stage. Thus, using *TP53* mutations as diagnostic markers of HGSC will most likely require next generation sequencing (NGS) with high precision for rare mutations in small amounts of available DNA.

In liquid biopsies, cancer‐specific information may be found in cell‐free DNA (cfDNA). Detection of mutations in circulating tumor DNA (ctDNA) from plasma for diagnostic purposes significantly increases specificity to 100%.^6^, ^7^, ^8^ Other liquid biopsies suggested for diagnostic testing include cervical smears,^3^, ^7^, ^9^ intrauterine brush or lavage,^7^, ^10^ as well as ovarian cyst‐^11^ and ascites fluid.^12^ However, a comparative study of the usability of *TP53* mutations has not been conducted using multiple proximal biopsies and primary tumor samples from the same patient.^3^, ^7^, ^8^, ^9^, ^10^, ^11^, ^12^ It remains unclear whether ctDNA in liquid biopsies can replace a tumor biopsy in the preoperative diagnostic work‐up, and whether detected mutations in plasma ctDNA are identical to the clonal variants detected in other compartments near the primary tumor.

The aim of this proof‐of‐concept study was to enhance the detection of somatic *TP53* variants in liquid and solid biopsies from patients with HGSC. We developed a highly sensitive and robust *TP53* mutation panel to detect somatic *TP53* variants with the simple, multiplexed, PCR‐based barcoding of DNA for sensitive mutation detection using sequencing (SiMSen‐seq) technique.^13^ We analyzed 94 samples from 11 patients diagnosed with HGSC, including primary tumors, plasma, ascites, vaginal and both cell pellet (S) and liquid phase (LB) from ovarian cyst fluids, endocervical and endometrial samples.

## 2 MATERIALS AND METHODS

### 2.1 Patient selection, sample collection and DNA extraction

This study included patients diagnosed with HGSC between 03‐2016 and 12‐2016 at Sahlgrenska University Hospital (Gothenburg, Sweden). The inclusion criteria were (1) suspected advanced OC (stage III‐IV) and (2) peritoneal carcinomatosis, ascites, and/or ovarian cystic formations with papillary structures or multilocular‐solid tumors visible on transvaginal ultrasound or computer tomography (CT) and admitted to hospital for surgical intervention. The exclusion criteria were (1) history of neoadjuvant chemotherapy, (2) prior surgery involving salpingectomy, oophorectomy, or sterilization, and (3) inability to comprehend information in Swedish, orally or written. Clinical specimens were collected on the day of curative surgery. Histopathology evaluation was performed by a board‐certified pathologist specializing in gynecological malignancies and only HGSC was included (Table S1). Genomic DNA was extracted from primary tumors, liquid compartments and cell pellets using Qiagen AllPrep DNA/RNA Mini Kit (Qiagen; Hilden, Germany), QIAamp Circulating Nucleic Acid Kit (Qiagen) and the QIAamp DNA Micro Kit (Qiagen) (Supplementary Material and Methods). For vaginal samples, DNA was eluted from the FTA card with four 3.5 mm punches by heating in deionized water.^14^

### 2.2 *TP53* ‐gene panel construction, library generation and data analysis

The presence of *TP53* mutations was evaluated using the simple, multiplexed, PCR‐based barcoding of DNA for sensitive mutation detection using sequencing (SiMSen‐seq) protocol,^13^ an ultra‐sensitive sequencing method with a detection limit of 0.1%. The *TP53* panel, designed as two subpanels of a total of 17 non‐overlapping assays, covers 618 nucleotide positions, representing hotspot mutations in exons 4–11, and provides comprehensive coverage of the DNA binding domain (amino acids 102–292; Table S2). Quantitative PCR was conducted on pooled libraries using the NEBNext Library Quant Kit for accurate quantification. Single‐end sequencing was executed on an Illumina platform (MiniSeq; NextSeq 550; Illumina, San Diego, CA) in 1× 150 base pairs mode with 20% PhiX (Illumina). Sequencing data were processed using the UMIErrorCorrect pipeline (version 0.24)^15^ (see Supplementary Material and Methods). The background error rate was calculated for each specimen type by dividing non‐reference nucleotides by corresponding UMI count at each position, excluding positions with called mutations. UMI counts were adjusted for barcode cycles^16^ and variable DNA input (Figure S1). The estimation of ctDNA molecules/ml sample, was calculated considering the mutated allele count and the volume of sample used during the SiMSen‐seq library generation process, specifically quantifying the fraction of DNA utilized relative to the total DNA extracted from the liquid sample. Tumor cell fraction was estimated using the VAF of the *TP53* variant, based on the two‐hit hypothesis^17^ and loss of heterozygosity in ovarian cancer.^18^, ^19^ Furthermore, to assess the applicability of the *TP53* panel, it was applied to two external datasets, Catalogue of Somatic Mutations in Cancer (COSMIC) database and CancerSEEK.^6^

## 3 RESULTS

### 3.1 Patient characteristics and samples

Eleven patients with HGSC stage IIB‐IVB, were included in the study (Table S1). DNA was extracted from 94 samples collected at seven different anatomical locations. Analyzed samples included: primary tumors (*n* = 11), ascites (*n* = 10), ovarian cyst fluid LB (*n* = 8), ovarian cyst fluid S (*n* = 8), plasma (*n* = 8), endocervical LB (*n* = 11), endocervical S (*n* = 11), endometrial LB (*n* = 9), endometrial S (*n* = 10), and vaginal samples (*n* = 8). Of the 11 patients, three had all sample types, four had nine, and the remaining four had between six and eight sample types (Figure S1).

### 3.2 Assessment of the sample types and technical evaluation of the *TP53* panel

**FIGURE 1:**
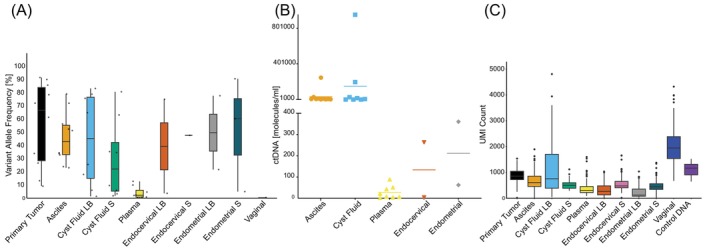
Assessment of *TP53* mutations in HGSC biopsies. (A) Distribution of VAF [%] for pathogenic variants across different sample types. (B) Detectable ctDNA for liquid samples. Median allele count per ml: Ascites 7523, cyst fluid LB 11717, plasma 14, endocervical LB 134, and endometrial LB 211. (C) UMI counts across sample types. LB, Liquid biopsy; S, Solid cell pellet; UMI, unique molecular identifiers.

A total of 15 unique mutations were detected in all samples examined, of which 13 (87%) were annotated as pathogenic, and the remaining two were classified as variants of unknown significance (VUS). The median VAF for pathogenic variants across all sample types was 33% (range, 0.2–91; Figure 1A); for all observed mutations, the median VAF was 30% (range, 0.2–91). The median VAF for VUS alone was 5% (range, 0.4–56; Figure S2A). High VAF mutations identified in the LBs of patients 7, 10, 13 and 20 were confirmed to be somatic (Figure S2B). The median ctDNA allele count for all LBs was 2195 molecules/ml (range, 1.7–952,521; Table S3), corresponding to a ctDNA fraction of 6% (range, 0.1–45). The cyst fluids had the largest amount of ctDNA among the LBs, with a median of 11,717 molecules/ml (range, 1061–952,521), while plasma had the lowest amount of ctDNA, with a median at 14 molecules/ml (range, 1.7–89; Figure 1B). The median tumor cell fraction across solid samples was 52% (range, 0.5–96), with primary tumors showing the highest median tumor fraction at 80% (range, 17–96; Figure S3 and Table S3).

The *TP53* panel's effectiveness was evaluated through dilution series with control DNA at concentrations of 100, 20, 10, and 5 ng. High proficiency of the panel was confirmed with distinct electropherogram peaks for all concentrations (Figure S4A,B). The panel demonstrated effective performance even with DNA input of <5 ng, and all assays performed adequately across all sample types (Figure S4D) with a low error rate (0.002%; Figure S4C). The mean sequencing UMI count was highest for vaginal samples (2037), followed by cyst fluid LB (1096), primary tumor (873), ascites (679), endocervical S (539), cyst fluid S (512), endometrial S (485), plasma (383), endocervical LB (321), and endometrial LB (225) in comparison to the mean sequencing UMI count for control DNA at 1156 (Figure 1C).

### 3.3 Concomitant mutations identified in paired samples from different anatomical locations

**FIGURE 2:**
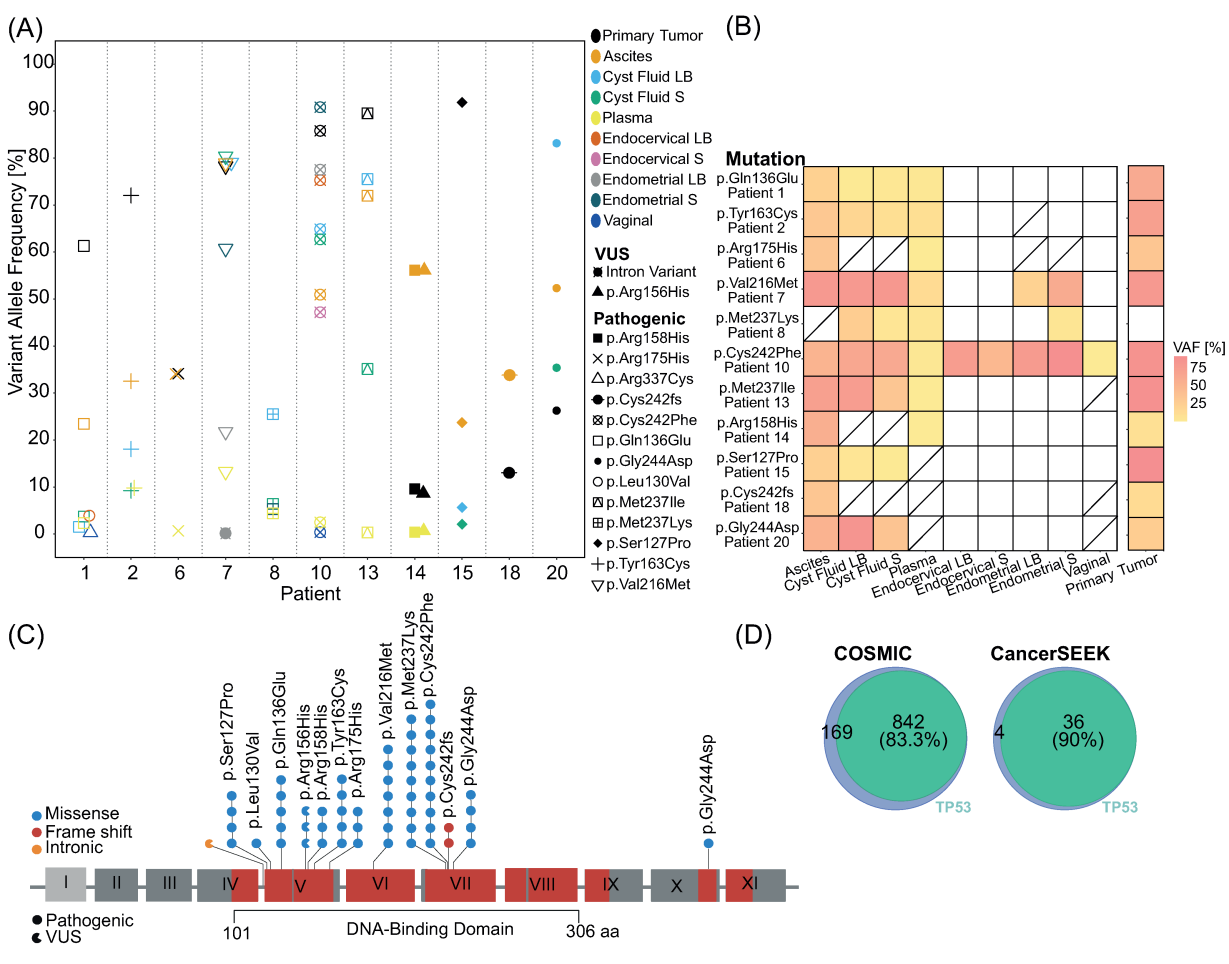
Comparative analysis and diagnostic efficacy of *TP53* mutations in different sample types. (A) Somatic variants identified in paired samples. Color annotates sample type. Symbol marks specific mutations. (B) Heatmap depicting the frequency of pathogenic mutations common with primary tumor or plasma across different sample types. Color intensity indicating VAF [%]. (C) Schematic *TP53*‐panel coverage (red) and mutations across the cohort. Lollipops show the distribution of mutations. Shape annotates clinical relevance; full indicates a pathogenic mutation, and open circle a VUS. Color annotates type of mutation: Blue, missense; red, deletion; orange, intronic. Each head represents a sample with the specific mutation. (D) The *TP53* panel covers 83.3% of COSMIC and 90% of CancerSEEK ovarian cancer mutations. LB, Liquid biopsy; Not available; S, Solid cell pellet.

All (11/11) patients exhibited a unique patient‐specific pathogenic mutation (median VAF 33%) which was observed in 2–10 of the paired samples (Figure 2A). Ten of 11 patients (91%) displayed the pathogenic mutation in three or more anatomical locations. The pathogenic mutation p.Cys242Phe was detected in all sample types in patient 10.

The primary tumor exhibited a pathogenic mutation in 91% (10/11) of the patients, median VAF 67% (range, 9–91). Among the various sample types analyzed, ascites, ovarian cyst fluid (LB + S) and plasma exhibited the highest number of shared variants with the primary tumor, each matching 100% of the observed mutations (10/10, 7/7, and 7/7, respectively; Figure 2B). In addition to the pathogenic mutation (p.Arg158His), patient 14 displayed a VUS (p.Arg156His; median VAF 5%; Table 1 and Figure S2A) on the same DNA molecule in the primary tumor, plasma, and ascites samples. Of the 15 unique *TP53* mutations, 13 were missense substitutions, one was a deletion, and one was an intron variant. As captured by eight (8/17) assays, the observed mutations were located in exon 5–7 (Figure 2C), except for one mutation in exon 10 (patient 1). The pathogenic mutations detected were p.Ser127Pro, p.Leu130Val, p.Gln136Glu, p.Arg158His, p.Tyr163Cys, p.Arg175His, p.Val216Met, p.Met237Ile, p.Met237Lys, p.Cys242Phe, p.Cys242fs, p.Gly244Asp, and p.Arg337Cys.

**TABLE 1:** The *TP53* mutations found in clinical samples from different anatomical regions.

Patient	Sample	Chr 17 base position	Base	Amino acid	VAF [%]	UMI count	Clinical relevance
1	Primary tumor	7,675,206	c.406C>G	p.Gln136Glu	61.33	1694	Pathogenic
1	Ascites	7,675,206	c.406C>G	p.Gln136Glu	23.04	6592	Pathogenic
1	Cyst fluid LB	7,675,206	c.406C>G	p.Gln136Glu	1.40	6664	Pathogenic
1	Cyst fluid S	7,675,206	c.406C>G	p.Gln136Glu	3.28	5580	Pathogenic
1	Plasma	7,675,206	c.406C>G	p.Gln136Glu	2.06	2623	Pathogenic
1	Endocervical LB	7,675,224	c.388C>G	p.Leu130Val	3.66	1392	Pathogenic
1	Vaginal	7,670,700	c.1009C>T	p.Arg337Cys	0.27	5520	Pathogenic
2	Primary tumor	7,675,124	c.488A>G	p.Tyr163Cys	72.09	1376	Pathogenic
2	Ascites	7,675,124	c.488A>G	p.Tyr163Cys	32.83	3277	Pathogenic
2	Cyst fluid LB	7,675,124	c.488A>G	p.Tyr163Cys	18.03	3650	Pathogenic
2	Cyst fluid S	7,675,124	c.488A>G	p.Tyr163Cys	9.57	4192	Pathogenic
2	Plasma	7,675,124	c.488A>G	p.Tyr163Cys	9.97	1574	Pathogenic
6	Primary tumor	7,675,088	c.524G>A	p.Arg175His	33.88	1830	Pathogenic
6	Ascites	7,675,088	c.524G>A	p.Arg175His	34.39	11,005	Pathogenic
6	Plasma	7,675,088	c.524G>A	p.Arg175His	0.33	10,188	Pathogenic
7	Primary tumor	7,674,885	c.646G>A	p.Val216Met	78.51	2262	Pathogenic
7	Ascites	7,674,885	c.646G>A	p.Val216Met	79.02	7093	Pathogenic
7	Cyst fluid LB	7,674,885	c.646G>A	p.Val216Met	78.90	4421	Pathogenic
7	Cyst fluid S	7,674,885	c.646G>A	p.Val216Met	80.71	4587	Pathogenic
7	Plasma	7,674,885	c.646G>A	p.Val216Met	12.78	2746	Pathogenic
7	Endometrial LB	7,674,885	c.646G>A	p.Val216Met	21.69	770	Pathogenic
7	Endometrial LB	7,675,239	c.376‐3C>A	Intron Variant	0.40	1260	VUS
7	Endometrial S	7,674,885	c.646G>A	p.Val216Met	60.35	2948	Pathogenic
8	Cyst fluid LB	7,674,253	c.710 T>A	p.Met237Lys	25.58	5140	Pathogenic
8	Cyst fluid S	7,674,253	c.710 T>A	p.Met237Lys	6.00	5469	Pathogenic
8	Plasma	7,674,253	c.710 T>A	p.Met237Lys	4.65	3310	Pathogenic
8	Endometrial S	7,674,253	c.710 T>A	p.Met237Lys	4.98	3677	Pathogenic
10	Primary tumor	7,674,238	c.725G>T	p.Cys242Phe	85.74	1767	Pathogenic
10	Ascites	7,674,238	c.725G>T	p.Cys242Phe	51.32	7508	Pathogenic
10	Cyst fluid LB	7,674,238	c.725G>T	p.Cys242Phe	64.89	3466	Pathogenic
10	Cyst fluid S	7,674,238	c.725G>T	p.Cys242Phe	63.12	4968	Pathogenic
10	Plasma	7,674,238	c.725G>T	p.Cys242Phe	2.33	4114	Pathogenic
10	Endocervical LB	7,674,238	c.725G>T	p.Cys242Phe	75.00	5313	Pathogenic
10	Endocervical S	7,674,238	c.725G>T	p.Cys242Phe	47.64	6433	Pathogenic
10	Endometrial LB	7,674,238	c.725G>T	p.Cys242Phe	77.69	4756	Pathogenic
10	Endometrial S	7,674,238	c.725G>T	p.Cys242Phe	90.61	5219	Pathogenic
10	Vaginal	7,674,238	c.725G>T	p.Cys242Phe	0.37	31,860	Pathogenic
13	Primary tumor	7,674,252	c.711G>T	p.Met237Ile	90.01	1511	Pathogenic
13	Ascites	7,674,252	c.711G>T	p.Met237Ile	72.02	6619	Pathogenic
13	Cyst fluid LB	7,674,252	c.711G>T	p.Met237Ile	75.76	4159	Pathogenic
13	Cyst fluid S	7,674,252	c.711G>T	p.Met237Ile	34.65	6560	Pathogenic
13	Plasma	7,674,252	c.711G>T	p.Met237Ile	0.75	1860	Pathogenic
14	Primary tumor	7,675,139	c.473G>A	p.Arg158His	9.139	1685	Pathogenic
14	Primary tumor	7,675,145	c.467G>A	p.Arg156His	9.145	1684	VUS
14	Ascites	7,675,139	c.473G>A	p.Arg158His	56.25	8154	Pathogenic
14	Ascites	7,675,145	c.467G>A	p.Arg156His	56.25	8154	VUS
14	Plasma	7,675,139	c.473G>A	p.Arg158His	0.85	2474	Pathogenic
14	Plasma	7,675,145	c.467G>A	p.Arg156His	0.85	2474	VUS
15	Primary tumor	7,675,233	c.379 T>C	p.Ser127Pro	91.49	1527	Pathogenic
15	Ascites	7,675,233	c.379 T>C	p.Ser127Pro	23.98	4471	Pathogenic
15	Cyst fluid LB	7,675,233	c.379 T>C	p.Ser127Pro	6.06	3926	Pathogenic
15	Cyst fluid S	7,675,233	c.379 T>C	p.Ser127Pro	1.79	5192	Pathogenic
18	Primary tumor	7,674,240	c.723del	p.Cys242fs	13.26	2029	Pathogenic
18	Ascites	7,674,240	c.723del	p.Cys242fs	33.77	4821	Pathogenic
20	Primary tumor	7,674,232	c.731G>A	p.Gly244Asp	26.61	2345	Pathogenic
20	Ascites	7,674,232	c.731G>A	p.Gly244Asp	52.36	5794	Pathogenic
20	Cyst fluid LB	7,674,232	c.731G>A	p.Gly244Asp	83.23	3388	Pathogenic
20	Cyst fluid S	7,674,232	c.731G>A	p.Gly244Asp	35.30	6238	Pathogenic

*Note*: Nucleotide and amino acid positions are numbered according to full‐length *TP53* reference genome hg38.

Abbreviations: del, deletion; S, solid cell pellet, LB, liquid biopsy; VAF, variant allele frequency.

### 3.4 The diagnostic potential of the *TP53* panel in different sample types

Considering the detection of a pathogenic mutation as a positive result, the samples were evaluated for their potential to support treatment decisions. The *TP53* panel detected a pathogenic mutation in 100% of the plasma (8/8), cyst fluid (8/8) and ascites (10/10) samples (Table 1). The plasma showed an 88% mutational commonality with the primary tumor (7/8), and 8/8 mutations were identified in additional ≥2 paired sample types (Figure 2B). Among the various sample types analyzed, ascites and ovarian cyst fluid LB + S exhibited the highest number of shared amino acid changes with plasma, each matching 100% of possible mutations (7/7 and 6/6, respectively). Positive results were less prominent in vaginal (25%; 2/8), endocervical (14%; 3/22) and endometrial (26%; 5/19) samples. For further evaluation of its theoretical clinical guidance potential, the *TP53* panel was applied to two external datasets. The panel covered 83% of the HGSC somatic mutations cataloged in the COSMIC database. Similarly, when applied to data from the CancerSEEK *TP53*‐mutated ovarian cancer primary tumors,^6^ 90% of the mutations were captured by the *TP53* panel (Figure 2D).

## 4 DISCUSSION

In this proof‐of‐concept study, we describe a highly sensitive *TP53* mutation panel that demonstrates technical reproducibility applicable to a variety of clinical samples from different anatomical locations. In total, 94 samples including the HGSC primary tumors, tumor proximal liquid biopsies, and cell pellets were analyzed using our in‐house developed *TP53* panel with a PCR‐based UMI approach for NGS. The results emphasize the significance of *TP53* alterations as a key molecular feature in HGSC. In all cases (11/11), a patient‐specific pathogenic *TP53* mutation was observed in more than one clinically relevant sample, which is consistent with previous observations of HGSC having one unique *TP53* driver mutation.^20^, ^21^ Somatic *TP53* mutations in HGSC have been reported across all coding regions^21^, ^22^; however, a significant enrichment of mutations was found in the DNA binding part of *TP53* (exons 5–7), aligning with the previously described prevalence of these specific *TP53* mutations.^2^ The detection of a unique pathogenic mutation per patient demonstrates the ability of the *TP53* panel to capture the heterogeneity of OC. Moreover, although considered to be a low‐penetrance mutation on its own, the VUS p.Arg156His may have a phenotype‐enhancing effect when co‐presenting with a second *TP53* mutation.^23^

Methods integrating protein and genetic biomarkers have shown varied effectiveness across different stages of cancer, with a notably high sensitivity of 98% for detecting advanced‐stage OC in plasma.^6^ In line, our results confirm that the identification of ctDNA in plasma is a good diagnostic tool that reflects the mutational burden of the primary tumor, as all plasma samples displayed a pathogenic mutation. The occurrence of ctDNA in non‐blood samples collected near the ovarian tumor has previously been shown,^7^, ^8^ offering unique advantages such as higher ctDNA to cfDNA ratios in more localized sampling. The *TP53* mutation panel detected pathogenic mutations in 100% of the ascites and ovarian cyst fluid samples, corresponding to 100% mutational concordance observed for both ascites and cyst fluid to paired plasma samples. Furthermore, in one patient, where no pathogenic mutation was detected in the primary tumor, the liquid biopsies displayed a common pathogenic mutation. This indicates that liquid biopsies may be an alternative to tumor biopsies for differential diagnostic purposes in cancers with high intra‐tumor heterogeneity. There is an emerging role for ascites ctDNA detection in outlining the genetic alterations of OC and highlights its potential for guiding treatment decisions such as neo‐adjuvant chemotherapy or primary debulking surgery.^8^, ^12^, ^19^ Although OC cyst fluids demonstrate impressive diagnostic potential for evaluating mutational profiles in ctDNA,^11^ this sample cannot be considered non‐invasive and fine needle aspirations for diagnostic purposes are not recommended.

Analysis of the mutation profile of endocervical samples on slides has shown diagnostic value for the detection of OC with droplet digital polymerase chain reaction and shallow whole genome sequencing.^3^, ^24^ However, the handling of this sample type is of great importance for DNA integrity^25^ as the use of methanol preservative solution could potentially contribute to DNA degradation and a lower detection rate. The endocervical and endometrial samples, all collected in methanol preservative solution, exhibited a low number of mutations, generally at lower VAF attributed to the low ratio of ctDNA. Also, low UMI counts were obtained for three endocervical liquid phase samples, and insufficient sequencing results were observed for three endometrial liquid phase samples.

We have shown that the SiMSen‐seq UMI method enabled detection of *TP53* mutations under challenging conditions. The utilization of UMIs allowed for reduction of sequencing errors by generating consensus reads for DNA molecules with the same UMI, correcting polymerase‐induced errors and minimizing background noise.^15^ In line, the background error rate was low (0.002%), underlining the reliability of the panel and methodology. This approach enhanced the potential to detect low‐frequency *TP53* mutations, even at minimal DNA input, <5 ng. However, though the study encompassed 94 samples, the included cohort is relatively small (*n* = 11), which limits the generalizability of the findings. Additionally, the *TP53* panel has been developed for the detection of HGSC hotspot mutations. In cases where the tumor presents with an atypical mutation, the absence of full gene coverage becomes a limitation. This proof‐of‐concept study has exclusively considered samples from HGSC; however, the data presented suggest that the panel could be applied to equivalent liquid biopsies from patients with other cancer types that exhibit a high frequency of *TP53* mutations. The method offers the flexibility to incorporate assays, thus accommodating the specific needs of the tumor type of interest.

In conclusion, this proof‐of‐concept study presents a promising *TP53* mutation panel capable of identifying mutations with low DNA input across various sample types. The findings suggest that *TP53* mutations associated with primary tumors can be detected in cells and liquid biopsies from ascites, cyst fluids, plasma, vaginal, endocervical, and endometrial samples.

## AUTHOR CONTRIBUTIONS

**Amanda Olsson Widjaja:** Methodology; investigation; validation; visualization; data curation; writing – original draft; writing – review and editing. **Peter Micallef:** Investigation; writing – review and editing. **Maria Lycke:** Resources; writing – review and editing. **Tobias Österlund:** Methodology; investigation; software; data curation; writing – review and editing. **Manuel Luna Santamaría:** Methodology; software; data curation; writing – review and editing. **Julia Hedlund Lindberg:** Investigation; resources; writing – review and editing. **Therese Carlsson:** Investigation; writing – review and editing. **Ulf Gyllensten:** Resources; writing – review and editing. **Anders Ståhlberg:** Supervision; funding acquisition; writing – review and editing. **Benjamin Ulfenborg:** Data curation; writing – review and editing; supervision. **Anna Linder:** Conceptualization; project administration; supervision; funding acquisition; writing – original draft; writing – review and editing. **Karin Sundfeldt:** Conceptualization; project administration; supervision; resources; funding acquisition; writing – original draft; writing – review and editing.

## FUNDING INFORMATION

The present study has been funded by the Sjöberg Foundation (2021‐01145 to Karin Sundfeldt), Swedish state under the agreement between the Swedish government and the county councils, the ALF‐agreement (965552 to Karin Sundfeldt), Cancera and the Swedish Cancer Society (21‐1848 to Karin Sundfeldt), the Assar Gabrielsson's Foundation (FB22‐67, and FB23‐118 to Anna Linder; FB22‐79, and FB23‐66 to Amanda Olsson Widjaja), the Lions Cancerfond Väst (2022 to Anna Linder), Nilsson‐Ehle Endowments (2022 to Anna Linder), Gunvor och Ivan Svenssons stiftelsen till minne av deras son Ivan (2023, and 2024 to Anna Linder), and Kungl. Vetenskaps‐och Vitterhets‐Samhället (KVVS) (2020 to Anna Linder). Anders Ståhlberg is funded by Region Västra Götaland; Swedish Cancer Society [22‐2080]; Swedish Research Council [2021‐01008]; the Swedish state under the agreement between the Swedish government and the county councils, the ALF‐agreement [965065]; Sweden's Innovation Agency [2020‐04141], and the Sjöberg Foundation.

## CONFLICT OF INTEREST STATEMENT

Anders Ståhlberg is co‐inventor of the SiMSen‐Seq technology that is patent protected (U.S. Serial No.:15/552,618). Anders Ståhlberg declares stock ownership in Tulebovaasta, Iscaff Pharma and SiMSen Diagnostics, and is a board member of Tulebovaasta. The other co‐authors declare no potential conflicts of interest.

## ETHICS STATEMENT

The study was performed according to Helsinki declaration and approved by the Swedish Ethical Review Authority (registration No. 510‐13 and 201‐15). All participants provided their written informed consent.

## Supporting information


**TABLE S1.** Patient characteristics.
**TABLE S2**. *TP53* assays.
**TABLE S3**. Sample and DNA overview.
**TABLE S4**. Sequencing coverage.
**FIGURE S1**. Overview, sample and *TP53* library set‐up.
**FIGURE S2**. VAF for VUS and somatic control validation.
**FIGURE S3**. Tumor cell fraction solid biopsies.
**FIGURE S4**. *TP53*‐panel and sample evaluation.

## Data Availability

All data generated or analyzed during this study are included in this published article (and its supplementary information files). Further information is available from the corresponding author upon request.

## References

[1] Steinberga I , Jansson K , Sorbe B . Quality indicators and survival outcome in stage IIIB‐IVB epithelial ovarian cancer treated at a single institution. In Vivo. 2019;33:1521‐1530. 31471400 10.21873/invivo.11632 PMC6755028

[2] Tuna M , Ju Z , Yoshihara K , Amos CI , Tanyi JL , Mills GB . Clinical relevance of TP53 hotspot mutations in high‐grade serous ovarian cancers. Br J Cancer. 2020;122:405‐412. 31780779 10.1038/s41416-019-0654-8 PMC7000721

[3] Paracchini L , Pesenti C , Delle Marchette M , et al. Detection of TP53 clonal variants in Papanicolaou test samples collected up to 6 years prior to high‐grade serous epithelial ovarian cancer diagnosis. JAMA Netw Open. 2020;3:e207566. 32609349 10.1001/jamanetworkopen.2020.7566 PMC7330718

[4] Piek JMJ . Dysplastic changes in prophylactically removed fallopian tubes of women predisposed to developing ovarian cancer. J Pathol. 2001;195:451‐456. 11745677 10.1002/path.1000

[5] Kuhn E , Kurman RJ , Vang R , et al. TP53 mutations in serous tubal intraepithelial carcinoma and concurrent pelvic high‐grade serous carcinoma‐evidence supporting the clonal relationship of the two lesions. J Pathol. 2012;226:421‐426. 21990067 10.1002/path.3023 PMC4782784

[6] Cohen JD , Li L , Wang Y , et al. Detection and localization of surgically resectable cancers with a multi‐analyte blood test. Science. 2018;359:926‐930. 29348365 10.1126/science.aar3247 PMC6080308

[7] Wang Y , Li L , Douville C , et al. Evaluation of liquid from the Papanicolaou test and other liquid biopsies for the detection of endometrial and ovarian cancers. Sci Transl Med. 2018;10:eaap8793. 29563323 10.1126/scitranslmed.aap8793 PMC6320220

[8] Han M‐R , Lee SH , Park JY , et al. Clinical implications of circulating tumor DNA from ascites and serial plasma in ovarian cancer. Cancer Res Treat. 2020;52:779‐788. 32106643 10.4143/crt.2019.700 PMC7373868

[9] Arildsen NS , Martin de la Fuente L , Masback A , et al. Detecting TP53 mutations in diagnostic and archival liquid‐based Pap samples from ovarian cancer patients using an ultra‐sensitive ddPCR method. Sci Rep. 2019;9:15506. 31664085 10.1038/s41598-019-51697-6 PMC6820715

[10] Maritschnegg E , Heitz F , Pecha N , et al. Uterine and tubal lavage for earlier cancer detection using an innovative catheter: a feasibility and safety study. Int J Gynecol Cancer. 2018;28:1692‐1698. 30376484 10.1097/IGC.0000000000001361 PMC6254778

[11] Wang Y , Sundfeldt K , Mateoiu C , et al. Diagnostic potential of tumor DNA from ovarian cyst fluid. Elife. 2016;5:1‐8. 10.7554/eLife.15175 PMC4946896 27421040

[12] Kfoury M , Hazzaz RE , Sanson C , et al. Circulating tumor DNA from ascites as an alternative to tumor sampling for genomic profiling in ovarian cancer patients. Biomark Res. 2023;11:1‐4. 37858195 10.1186/s40364-023-00533-1 PMC10588202

[13] Ståhlberg A , Krzyzanowski PM , Egyud M , Filges S , Stein L , Godfrey TE . Simple multiplexed PCR‐based barcoding of DNA for ultrasensitive mutation detection by next‐generation sequencing. Nat Protoc. 2017;12:664‐682. 28253235 10.1038/nprot.2017.006

[14] Gustavsson I , Lindell M , Wilander E , Strand A , Gyllensten U . Use of FTA card for dry collection, transportation and storage of cervical cell specimen to detect high‐risk HPV. J Clin Virol. 2009;46:112‐116. 19628427 10.1016/j.jcv.2009.06.021

[15] Österlund T , Filges S , Johansson G , Ståhlberg A . UMIErrorCorrect and UMIAnalyzer: software for consensus read generation, error correction, and visualization using unique molecular identifiers. Clin Chem. 2022;68:1425‐1435. 36031761 10.1093/clinchem/hvac136

[16] Sagitov S , Stahlberg A . Counting unique molecular identifiers in sequencing using a multi‐type branching process with immigration. J Theor Biol. 2023;558:111365. 36410451 10.1016/j.jtbi.2022.111365

[17] Knudson AG Jr . Mutation and cancer: statistical study of retinoblastoma. Proc Natl Acad Sci U S A. 1971;68:820‐823. 5279523 10.1073/pnas.68.4.820 PMC389051

[18] Godwin AK , Vanderveer L , Schultz DC , et al. A common region of deletion on chromosome 17q in both sporadic and familial epithelial ovarian tumors distal to BRCA1. Am J Hum Genet. 1994;55:666‐677. 7942844 PMC1918278

[19] Werner B , Powell E , Duggan J , et al. Cell‐free DNA from ascites identifies clinically relevant variants and tumour evolution in patients with advanced ovarian cancer. Mol Oncol. 2024;18:2668‐2683. 39115191 10.1002/1878-0261.13710 PMC11547227

[20] Cole AJ , Dwight T , Gill AJ , et al. Assessing mutant p53 in primary high‐grade serous ovarian cancer using immunohistochemistry and massively parallel sequencing. Sci Rep. 2016;6:26191. 27189670 10.1038/srep26191 PMC4870633

[21] Ahmed AA , Etemadmoghadam D , Temple J , et al. Driver mutations in TP53 are ubiquitous in high grade serous carcinoma of the ovary. J Pathol. 2010;221:49‐56. 20229506 10.1002/path.2696 PMC3262968

[22] Cancer Genome Atlas Research N . Integrated genomic analyses of ovarian carcinoma. Nature. 2011;474:609‐615. 21720365 10.1038/nature10166 PMC3163504

[23] Quesnel S , Verselis S , Portwine C , et al. p53 compound heterozygosity in a severely affected child with Li‐Fraumeni syndrome. Oncogene. 1999;18:3970‐3978. 10435620 10.1038/sj.onc.1202783

[24] Paracchini L , Mannarino L , Romualdi C , et al. Genomic instability analysis in DNA from Papanicolaou test provides proof‐of‐principle early diagnosis of high‐grade serous ovarian cancer. Sci Transl Med. 2023;15:1‐11. 10.1126/scitranslmed.adi2556 38055801

[25] Schumacher S , Malchau Lauesgaard J , Carlsson T , Linder A , Sundfeldt K . Optimization of pre‐analytical handling to maintain DNA integrity in diagnostic Papanicolaou tests. J Mol Diagn. 2025;27:199‐208. 39828035 10.1016/j.jmoldx.2024.12.008 PMC12179505

