# Supplementary material for: Detecting TP53 mutations in paired liquid and tissue biopsies from patients with high‐grade serous ovarian carcinoma
Source: Int J Cancer. 2025 Dec 9;158(8):2043–50. doi: 10.1002/ijc.70277 (PMC12922644; doi:10.1002/ijc.70277)
Supplement: Supplementary file 1 — TABLE S1. Patient characteristics. TABLE S2. TP53 assays. TABLE S3. Sample and DNA overview. TABLE S4. Sequencing coverage. FIGURE S1. Overview, sample and TP53 library set‐up. FIGURE S2. VAF for VUS and somatic control validation. FIGURE S3. Tumor cell fraction solid biopsies. FIGURE S4. TP53‐panel and sample evaluation. [file IJC-158-2043-s001.zip › Supporting Information.pdf]

Supporting Information for

Detecting *TP53* mutations in paired liquid and tissue biopsies from patients with high-grade serous ovarian carcinoma

Amanda Olsson Widjaja, Peter Micallef, Maria Lycke, Tobias Österlund, Manuel Luna Santamaría, Julia Hedlund Lindberg, Therese Carlsson, Ulf Gyllensten, Anders Ståhlberg, Benjamin Ulfenborg, Anna Linder, and Karin Sundfeldt

Table of content

Supplementary Material and Methods 1
Sample collection 1
TP53-gene panel construction and library generation 2
DNA extraction 3
Data analysis 3
Supplementary Tables 5
Supplementary Table S1 Patient Characteristics 5
Supplementary Table S2 *TP53* assays 6
Supplementary Table S3 Sample and DNA Overview 7
Supplementary Table S4 Sequencing coverage (Separate Excel file)
Supplementary Figures 11
Supplementary Figure S1. Overview, sample and *TP53* library set-up. 11
Supplementary Figure S2. VAF for VUS and somatic control validation 12
Supplementary Figure S3 Tumor cell fraction solid biopsies. 13
Supplementary Figure S4 *TP53*-panel and sample evaluation. 14
References supporting information 15

Supplementary Material and Methods

Sample collection

Vaginal samples were collected with a cotton swab and transferred onto FTA Elute Micro Cards (FTA, GE Healthcare, Cardiff, UK) the day of admission and stored in room temperature. After anesthesia but prior to midline incision plasma, endocervical and endometrial samples were acquired. To facilitate cell-free DNA isolation, blood was collected in preservative blood tubes

(BCT, Streck 2189629, La Vista, Nebraska, USA), followed by two centrifugations. Endocervical and endometrial samples were acquired with a cytobrush, one for each compartment and placed in separate methanol conservative solution (ThinPrep PreservCyt, Marlborough, Massachusetts, USA) at room temperature. The cytobrush used for collection of endometrial samples were customized to avoid contamination from the cervical canal (Tao brush, Indiana University Medical Center endometrial sampler, Cook OB/GYN, Indianapolis, Indiana, USA). Ascites was collected at the time of midline incision. Fluid from ovarian cysts was sampled immediately after its surgical removal. All samples were aliquoted, coded, and stored at -80°C. The handling and processing were standardized for all patients included in the study. The ovarian cyst fluid, endocervical, and endometrial samples were separated into a liquid biopsy phase (LB) and a solid cell pellet phase (S) before analysis.

TP53-gene panel construction and library generation

The presence of *TP53* mutations was evaluated using the Simple, Multiplexed, PCR-based barcoding of DNA for Sensitive mutation detection using Sequencing (SiMSen-seq) protocol [1], an ultra-deep targeted sequencing method that has a described detection limit of 0.1%. The *TP53* panel, designed as two subpanels of non-overlapping assays represent hotspot mutations in exons 4-11 and provide full coverage of the DNA binding domain (amino acids 102-292). Median amplicon length was 76 bp (range, 70-86). In-house designed primers were used with previously published primers [2]. Target primers with adequate specificity (with confirmed efficiency 95-105) were included in the final *TP53* panel (**Table S2**). Primary tumors (patient 1-11), ovarian cyst fluid LB (patient 2, 7 & 8), endocervical LB (patient 2, 7 & 8), endometrial LB (patient 7 & 8) underwent three barcode cycles (BC). All the other libraries were generated with five BCs. All libraries were amplified with 27 adapter PCR cycles. Library dilution series was prepared using control DNA (Human Genomic DNA, Roche Diagnostics GmbH, Mannheim, Germany) for panel evaluation, and the panels were verified with TapeStation

(Agilent Technologies, Santa Clara, CA, USA) analysis. Library PCR products underwent AMPure XP bead-based clean-up to remove non-specific PCR products, following manufacturer's protocol. Library concentrations were measured utilizing a 5200 Fragment Analyzer (Agilent) with a DNF-474 HS NGS Fragment Kit for a range of 100– 6000 base pairs. Quantitative PCR was conducted on pooled libraries using the NEBNext Library Quant Kit New England Biolabs, Ipswich, MA, USA) for accurate quantification. Single-end sequencing was executed on an Illumina platform (MiniSeq; NextSeq 550; Illumina, San Diego, CA, USA) in 1x150 base pairs mode with 20% PhiX (Illumina).

DNA extraction

Genomic DNA was extracted using Qiagen AllPrep DNA/RNA Mini Kit (Qiagen; Hilden, Germany) for primary tumor tissue, QIAamp Circulating Nucleic Acid Kit (Qiagen) for ascites, cyst fluid LB, plasma, endocervical LB, and endometrial LB, and the QIAamp DNA Micro Kit (Qiagen) for corresponding solid cell pellet (S). Median LB volume at extraction was 2 ml for ascites, ovarian cyst fluid LB, and plasma, 4 ml for endocervical LB, and 5 ml for endometrial LB (**Table S3**). For vaginal samples, DNA was eluted from four 3.5 mm diameter punches per patient by heating in deionized water [3]. For the germline reference, DNA was extracted from formalin-fixed and paraffin-embedded (FFPE) stroma tissue (Patient nr. 7, 10, 13 and 20) using the QIAamp DNA FFPE Tissue Kit (Qiagen). All extractions were carried out according to the manufacturer's instructions.

Data analysis

Sequencing data were processed using the UMIErrorCorrect pipeline (version 0.24) [4], implemented in Python (version 3.8.5). Reads were aligned to the hg38 reference genome utilizing the Burrows-Wheeler alignment tool, bwa mem [5]. Data were filtered by UMI family size cut-off ≥ 3. A variant quality score cutoff of 20 (Q20) was applied to the VCF-files. Consensus read depth and variant allele frequency (VAF) were applied with cutoff of 3 and

0.1%, respectively. Functional annotation of filtered VCF files was performed with SNPEff [6] and Variant Effect Predictor tool [7]. Variants confirmed as pathogenic or likely pathogenic were categorized under the clinical relevance classification *Pathogenic*. Three variants (p.Pro128His, p.Leu130Ile, and p.Tyr220His) were excluded from the analysis, as they were consistently found across all samples of the same type (LB or S) and thus considered likely artifacts.

The background error rate was calculated for each specimen type by dividing non-reference nucleotides by corresponding UMI count at each position, excluding positions with called mutations. UMI counts were adjusted for BCs [8] and variable DNA input (**Figure S1**). DNA yield was quantified with Qubit 1X dsDNA High Sensitivity Assay Kit (ThermoFisher Scientific, Waltham, MA, USA). The estimation of ctDNA molecules per milliliter sample, was calculated considering the mutated allele count and the volume of sample used during the SiMSen-seq library generation process, specifically quantifying the fraction of DNA utilized relative to the total DNA extracted from the liquid sample. The fraction of ctDNA was calculated based on assumed 310 haploid genome equivalents per ng DNA [9]. Tumor cell fraction was estimated using the VAF of the *TP53* variant, based on the two-hit hypothesis [10] and loss of heterozygosity in ovarian cancer [11, 12]. Furthermore, to assess the applicability of the *TP53* panel, it was applied to two external datasets, Catalogue of Somatic Mutations in Cancer (COSMIC) database and CancerSEEK [2], to calculate a theoretical mutational coverage.

Supplementary Tables

Supplementary Table S1 Patient Characteristics.

Patient	Smoking	Age at Diagnosis	Menopausal Status	Menopausal Age	CA-125	BMI	Histology	FIGO Stage
1	N	52	MP	NA	966	23	HGSC	IIIC
2	N	40	F	-	4514	21	HGSC	IIIC
6	N	57	MP	51	264	20	HGSC	IIIC
7	N	64	MP	50	887	21	HGSC	IIIC
8	N	67	MP	57	397	22	HGSC	IIIC
10	N	69	MP	54	1460	26	HGSC	IIIC
13	N	68	MP	50	1206	22	HGSC	IVA
14	N	58	MP	49	562	25	HGSC	IVB
15	N	48	F	-	569	24	HGSC	IIIC
18	N	71	MP	53	891	22	HGSC	IIIC
20	N	60	MP	50	1363	18	HGSC	IIB

Abbreviations: N, no; MP, menopause; F, fertile; NA, not available; CA-125, serum cancer antigen 125; BMI, body mass index; HGSC, high grade serous ovarian carcinoma; FIGO, The international federation of gynecology and obstetrics.

Supplementary Table S2 *TP53* assays.

Assay Name	Target Primer F	Target Primer R	Amplicon length (bp)	Exon
TP53 91 106	AAGAAGCCCAGACGGAAACC	GCACCAGCCCCCTCCT	86	4
TP53 113 125	CCCCTCAGGGCAACTGAC	AGCTACGGTTTCCGTCTGG	78	4
TP53 126 131	GGCCAGTTGGCAAAACATCT	GCCCTGACTTTCAACTCTGTCT	73	5
TP53 132 142	GAATCAACCCACAGCTGCAC	AGTACTCCCCTGCCCTCAAC	78	5
TP53 151 163	CGTCATGTGCTGTGACTGCTT	CAGCTGTGGGTTGATTCCA	81	5
TP53 166 179	TGGCCATCTACAAGCAGTCA	GAGCAGCGCTCATGGTG	70	5
TP53 188 195	CGCAAATTTCCTTCCACTCG	CTGATTCCTCACTGATTGCTCTT	71	6
TP53 208 218	TTGCGTGTGGAGTATTTGGA	AGACCTCAGGCGGCTCATAG	76	6
TP53 219 224	TAACCCCTCCTCCCAGAGAC	TTTTCGACATAGTGTGGTGGTG	78	6
TP53 233 245	TGGCTCTGACTGTACCACCA	ATGGGCCTCCGGTTCAT	78	7
TP53 248 261	GTGGCAAGTGGCTCCTGA	CATGGGCGGCATGAAC	76	7
TP53 262 267	AACACGCACCTCAAAGCTG	GCCTCTTGCTTCTCTTTTCCT	72	8
TP53 272 283	GGGACGGAACAGCTTTGAG	GCGGAGATTCTCTTCCTCTGT	76	8
TP53 298 306	AAAGGGGAGCCTCACCAC	ACCGCTTCTTGTCCTGCTT	71	8
TP53 323 331	CACTTGATAAGAGGTCCCAAGACTTA	CCCAGCCAAAGAAGAAACCA	77	9
TP53 334 343	TCCTCTGTTGCTGCAGATCC	TGAGTTCCAAGGCCTCATTC	74	10
TP53 368 374	TTTTATGGCGGGAGGTAGACT	TCATCTCTCCTCCCTGCTTCT	76	11

Abbreviations: Bp, Base pair; F, Forward; R, Reverse.

Supplementary Table S3 Sample and DNA Overview.

Sample	Patient	Allele Count	Total DNA (S) [ng]	Tumour DNA (S) [ng]	Original Sample Volume [mL]	ctDNA Conc. per mL sample [molecules/mL]	Tumor-cell (S) Fraction [%]
Primary Tumor	1	1039	37253.0	22848.8			76.0
	2	992	38344.0	27643.3			83.8
	6	620	19858.0	6727.8			50.6
	7	1776	40203.0	31564.8			88.0
	8		1618.0				
	10	1515	40365.0	34608.4			92.3
	13	1360	30044.0	27041.6			94.7
	14	154	11361.5	1038.7			16.7
	15	1397	49507.0	45292.3			95.6
	18	269	47648.5	6318.2			23.4
	20	624	9056.5	2409.9			42.0
	**Mean**	**974.6**	**29568.9**	**20549.4**			**66.3**
Cyst Fluid (S)	1	183	12275.0	4026			6.4
	2	401	9375.0	896.8			17.5
	6						
	7	3702	5200.0	4196.7			89.3
	8	328	1070.0	64.2			11.3
	10	3136	630.0	397.7			77.4
	13	2273	820.0	284.1			51.5
	14						
	15	93	2540.0	45.5			3.5
	18						
	20	2202	469.0	165.6			52.2
	**Mean**	**1539.8**	**4047.4**	**806.6**			**38.6**
Endocervical (S)	1		7408.9				
	2		327.2				
	6		244.7				
	7		14973.0				
	8		167.7				
	10	3065	371.5	177.0			64.5
	13		488.4				
	14		7686.0				
	15		105.4				
	18		7434.0				
	20		4226.9				
	**Mean**	**3065**	**3948.5**	**177.0**			**64.5**

Supplementary Table S3 Sample and DNA Overview continued (2/4).

Sample	Patient	Allele Count	Total DNALB [ng/ml] S [ng]	Tumour DNA (S) [ng]	Original Sample Volume [mL]	ctDNA Conc. per mL sample [molecules/mL]	Tumor-cell (S) Fraction [%]
**Endometrial (S)**	1		9300.0				
	2		2800.0				
	6						
	7	1779	1880.0	1134.5			75.3
	8	183	8905.0	443.2			9.5
	10	4729	28940.0	26222.9			95.1
	13		805.0				
	14		820.0				
	15		1070.0				
	18		2640.0				
	20		304.0				
	**Mean**	**2230.3**	**5746.4**	**9266.9**			**60.0**
**Vaginal**	1	15	32.8	0.1			0.5
	2		67.5				
	6		775.0				
	7		730.0				
	8		169.0				
	10	117	358.5	1.0			0.7
	13		13.2				
	14		1085.0				
	15		72.5				
	18						
	20						
	**Mean**	66	**367.1**	**0.6**			**0.6**

Sample	Patient	Allele Count	Total DNA LB per ml sample [ng/ml]	Tumour DNA (S) [ng]	Original Sample Volume [mL]	ctDNA Conc. per mL sample [molecules/mL]	ctDNA (LB) Fraction [%]
**Plasma**	1	54	8.8		2	6.2	0.2
	2	157	56.6		2	88.8	0.5
	6	34	21.4		2	7.3	0.1
	7	351	14.7		2	51.5	1.1
	8	154	13.8		2	21.2	0.5
	10	96	45.0		2	43.2	0.3
	13	14	91.0		2	1.7	0.1
	14	21	18.6		2	3.9	0.1
	15						
	18						
	20						
	**Mean**	**110.125**	**23.5**		**2**	**28.0**	**0.4**

Supplementary Table S3 Sample and DNA Overview continued (3/4).

Sample	Patient	Allele Count	Total DNA LB per ml sample [ng/ml]	Tumour DNA (S) [ng]	Original Sample Volume [mL]	ctDNA Conc. per mL sample [molecules/mL]	ctDNA (LB) Fraction [%]
**Ascites**	1	1519	189.8		2	2882.3	4.9
	2	1076	22997.6		2	247453.6	3.5
	6	3785	198.7		2	7521.6	12.2
	7	5605	532.9		2	29870.7	18.1
	8						
	10	3853	195.3		2	7524.7	12.4
	13	4767	217.4		2	10361.0	15.4
	14	4587	159.2		2	7300.4	14.8
	15	4587	93.8		2	4303.8	14.8
	18	1628	205.7		2	3349.1	5.3
	20	3034	395.4		2	11995.5	9.8
	**Mean**	**3444.1**	**2518.6**		**2**	**33256.3**	**11.1**
**Cyst Fluid LB**	1	93	29960.0		2	27862.8	0.3
	2	658	36190.0		2	952520.8	8.5
	6						
	7	3488	1415.9		2	197543.6	45.0
	8	1315	324.2		2	17053.8	17.0
	10	2249	47.2		2	1061.4	7.3
	13	3151	202.5		2	6379.6	10.2
	14						
	15	238	297.8		2	1507.9	1.6
	18						
	20	2820	46.7		2	1316.5	9.1
	**Mean**	**1751.5**	**8560.5**		**2**	**150655.8**	**12.4**
**Endocervical (LB)**	1	51	5.9		3	4.2	0.2
	2		3.4				
	6		4.1				
	7		1.9				
	8		4.5				
	10	3985	6.6		5	263.7	13.0
	13		5.7				
	14		28.8				
	15		2.7				
	18		11.2				
	20		14.6				
	**Mean**	**2018**	**8.1**		**4**	**134.0**	**6.5**

Supplementary Table S3 Sample and DNA Overview continued (4/4).

Sample	Patient	Allele Count	Total DNA LB per ml sample [ng/ml]	Tumour DNA (S) [ng]	Original Sample Volume [mL]	ctDNA Conc. per mL sample [molecules/mL]	ctDNA (LB) Fraction [%]
**Endometrial (LB)**	1		4.7				
	2		2.7				
	6						
	7	167	9.3		5	62.0	2.2
	8		2.9				
	10	3695	9.8		5	361.0	11.9
	13		2.9				
	14		4.5				
	15		2.4				
	18		8.8				
	20		2.3				
	**Mean**	**1931**	**5.0**				**7.0**

Abbreviations: LB, Liquid Biopsy; S, Solid cell pellet.

Supplementary Figures

		1	2	3	4	5	6	7	8	9	10
		TP53 Libraries									
	Patients	Primary Tumor	Ascites	Cyst Fluid LB	Cyst Fluid S	Plasma	Endocervical LB	Endocervical S	Endometrial LB	Endometrial S	Vaginal
1		1 10ng	1 10ng	1 10ng	1 10ng	1 7.7ng	1 7.1ng	1 10ng	1 8.7ng	1 10ng	1 2.6ng
2		2 10ng	2 10ng	2 12.5ng	2 10ng	2 10ng	2 8.6ng	2 10ng	NA	2 10ng	2 5.4ng
3		6 10ng	6 10ng	-	-	6 10ng	6 5.7ng	6 10ng	-	-	6 10ng
4		7 10ng	7 10ng	7 12.5ng	7 10ng	7 10ng	7 5.9ng	7 10ng	7 12.5ng	7 10ng	7 10ng
5		8 10ng	-	8 10ng	8 10ng	8 10ng	8 16.4ng	8 10ng	8 7.2ng	8 10ng	8 10ng
6		10 10ng	10 10ng	10 12.5ng	10 10ng	10 10ng	10 10ng	10 10ng	10 10ng	10 10ng	10 10ng
7		13 10ng	13 10ng	13 10ng	13 10ng	13 7.2ng	13 10ng	13 10ng	13 5.8ng	13 10ng	-
8		14 10ng	14 10ng	-	-	14 10ng	14 10ng	14 10ng	14 7.2ng	14 10ng	14 10ng
9		15 10ng	15 10ng	15 4.7ng	15 10ng	-	15 5.4ng	15 9.8ng	15 10ng	15 10ng	15 5.8ng
10		18 10ng	18 10ng	-	-	-	18 10ng	18 10ng	18 10ng	18 10ng	-
11		20 10ng	20 10ng	20 10ng	20 10ng	-	20 10ng	20 10ng	20 4.7ng	20 10ng	-

Supplementary Figure S1. Overview, sample and *TP53* library set-up.

X-axis annotates the number of sample types, Y-axis the number of patients. Patient study-number is indicated together with the DNA amount used per PCR-reaction for the specified sample.

Abbreviations: LB, Liquid biopsy; NA, Not analyzed; -, Not available; SiMSen-seq, Simple, Multiplexed, PCR-based barcoding of DNA for Sensitive mutation detection using Sequencing; S, Solid. Created with BioRender.com.

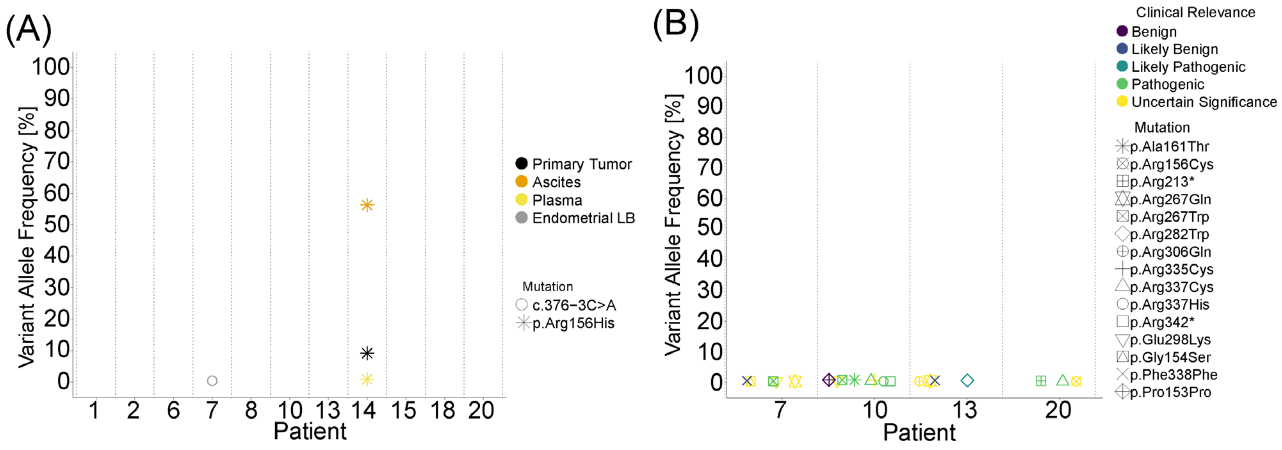

Supplementary Figure S2. VAF for VUS and somatic control validation.

Detection and confirmation of VUS and somatic mutations across sample types.

(A) All VUS detected across all sample types. (B) For germline reference, FFPE stroma tissue for four patients (nr. 7, 10, 13 and 20) were sequenced and high VAF [%] mutations in LBs for the patients were confirmed to be somatic. Color annotates sample type. Symbol marks specific mutations.

Abbreviations: FFPE, formalin-fixed and paraffin-embedded; LB, Liquid biopsy; VAF, Variant allele frequency; VUS, Variants with uncertain significance.

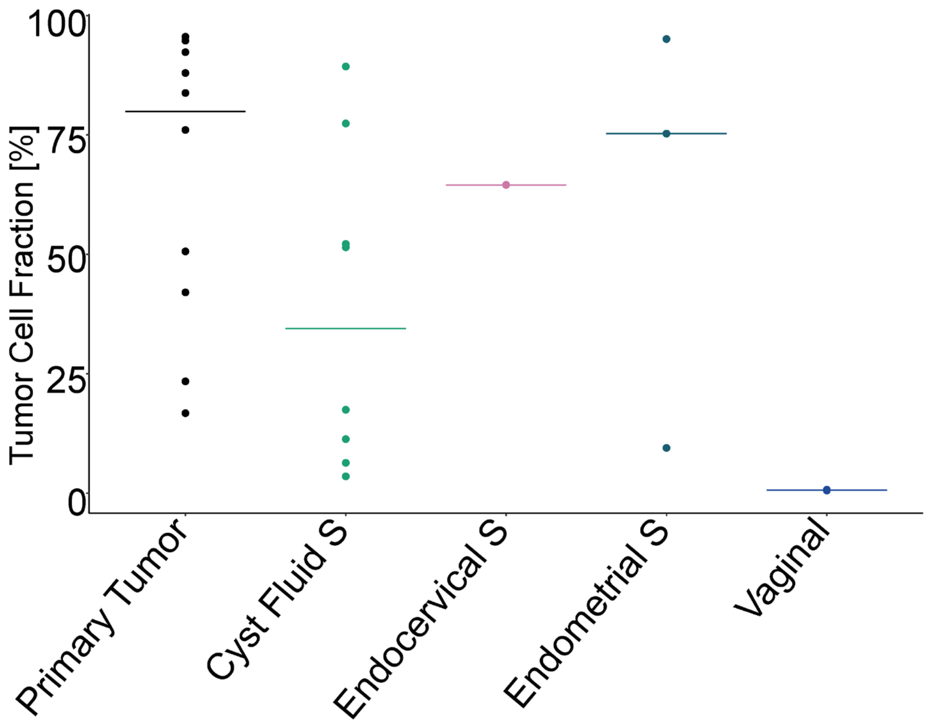

Supplementary Figure S3 Tumor cell fraction solid biopsies.

Tumor cell fractions across non-liquid sample types. Horizontal line annotates the median value. Median tumor cell fraction: primary tumor 80%, cyst fluid S 34%, endocervical S 65%, endometrial S 75%, and vaginal 0.6%.

Abbreviation: S, Solid cell pellet.

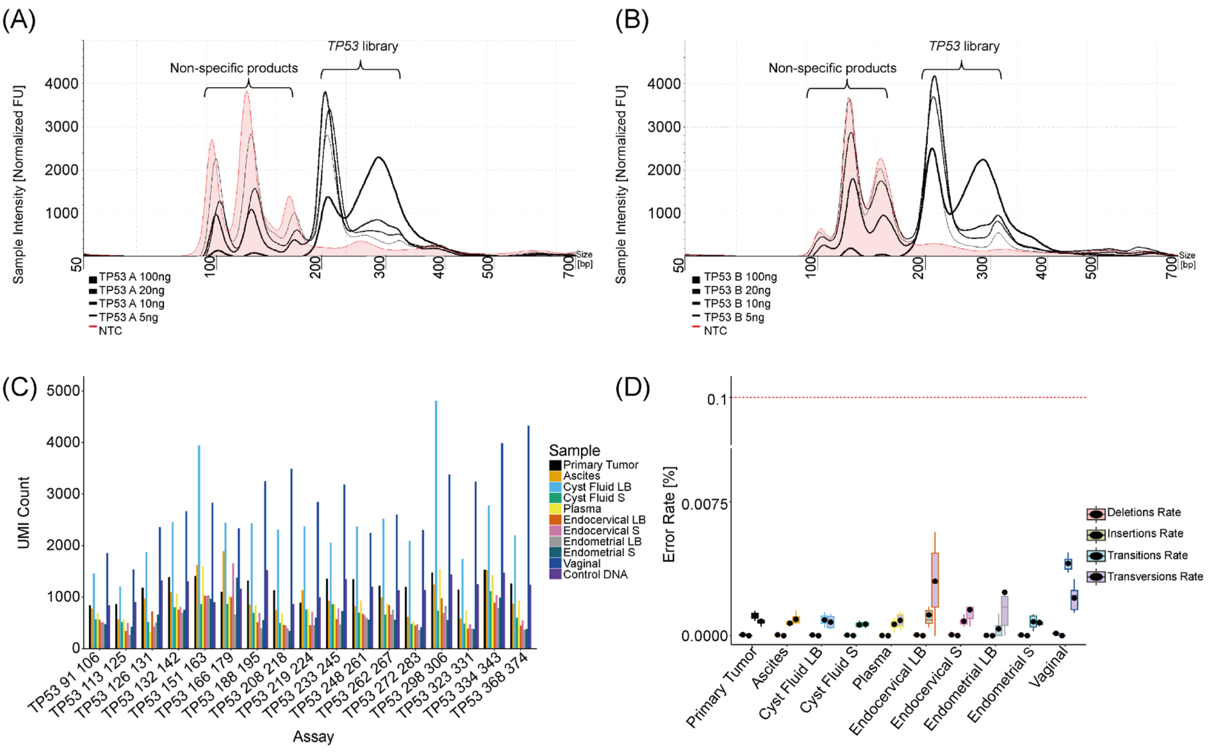

Supplementary Figure S4 *TP53*-panel and sample evaluation.

Technical assessment of *TP53* mutation detection sensitivity and sequencing UMI count. Electropherograms show control DNA dilution series with black gradients and NTC in red of subpanels A (A) and B (B). (C) Quantitative analysis of genome equivalents normalized to 10 ng of DNA using *TP53* assays across the different sample types. (D) Distribution of sample error rates for different mutation types. Dashed line annotates the 0.1% detection limit.

Abbreviations: FU, Fluorescence units; LB, Liquid biopsy; NTC, No-template control; SiMSen-seq, Simple, Multiplexed, PCR-based barcoding of DNA for Sensitive mutation detection using Sequencing; S, Solid cell pellet; UMI, unique molecular identifiers.

References supporting information

1. Ståhlberg A, Krzyzanowski PM, Egyud M, Filges S, Stein L, Godfrey TE. Simple multiplexed PCR-based barcoding of DNA for ultrasensitive mutation detection by next-generation sequencing. *Nature Protocols* 2017;**12**: 664-82.

2. Cohen JD, Li L, Wang Y, Thoburn C, Afsari B, Danilova L, Douville C, Javed AA, Wong F, Mattox A, Hruban RH, Wolfgang CL, et al. Detection and localization of surgically resectable cancers with a multi-analyte blood test. *Science* 2018;**359**: 926-30.

3. Gustavsson I, Lindell M, Wilander E, Strand A, Gyllensten U. Use of FTA card for dry collection, transportation and storage of cervical cell specimen to detect high-risk HPV. *J Clin Virol* 2009;**46**: 112-6.

4. Österlund T, Filges S, Johansson G, Ståhlberg A. UMIErrorCorrect and UMIAnalyzer: Software for Consensus Read Generation, Error Correction, and Visualization Using Unique Molecular Identifiers. *Clinical Chemistry* 2022;**68**: 1425-35.

5. Li H, Durbin R. Fast and accurate short read alignment with Burrows–Wheeler transform. *Bioinformatics* 2009;**25**: 1754-60.

6. Cingolani P, Platts A, Wang le L, Coon M, Nguyen T, Wang L, Land SJ, Lu X, Ruden DM. A program for annotating and predicting the effects of single nucleotide polymorphisms, SnpEff: SNPs in the genome of Drosophila melanogaster strain w1118; iso-2; iso-3. *Fly (Austin)* 2012;**6**: 80-92.

7. McLaren W, Gil L, Hunt SE, Riat HS, Ritchie GRS, Thormann A, Flicek P, Cunningham F. The Ensembl Variant Effect Predictor. *Genome Biology* 2016;**17**.

8. Sagitov S, Stahlberg A. Counting unique molecular identifiers in sequencing using a multi-type branching process with immigration. *J Theor Biol* 2023;**558**: 111365.

9. Piovesan A, Pelleri MC, Antonaros F, Strippoli P, Caracausi M, Vitale L. On the length, weight and GC content of the human genome. *BMC Res Notes* 2019;**12**: 106.

10. Knudson AG, Jr. Mutation and cancer: statistical study of retinoblastoma. *Proc Natl Acad Sci U S A* 1971;**68**: 820-3.

11. Godwin AK, Vanderveer L, Schultz DC, Lynch HT, Altomare DA, Buetow KH, Daly M, Getts LA, Masny A, Rosenblum N, et al. A common region of deletion on chromosome 17q in both sporadic and familial epithelial ovarian tumors distal to BRCA1. *Am J Hum Genet* 1994;**55**: 666-77.

12. Werner B, Powell E, Duggan J, Cortesi M, Lee YC, Arora V, Athavale R, Dean M, Warton K, Ford CE. Cell-free DNA from ascites identifies clinically relevant variants and tumour evolution in patients with advanced ovarian cancer. *Mol Oncol* 2024.
